# Melanoma molecular subtyping and scoring model construction based on ligand-receptor pairs

**DOI:** 10.3389/fgene.2023.1098202

**Published:** 2023-01-26

**Authors:** Zexu Lin, Xin Lin, Yuming Sun, Shaorong Lei, Gengming Cai, Zhexuan Li

**Affiliations:** ^1^ Department of Plastic and Cosmetic Surgery, Xiangya Hospital, Central South University, Changsha, Hunan Province, China; ^2^ Department of Plastic and Cosmetic Surgery, First Affiliated Hospital of Quanzhou, Fujian Medical University, Quanzhou, China; ^3^ Department of Otolaryngology-Head and Neck Surgery, First Affiliated Hospital of Quanzhou, Fujian Medical University, Quanzhou, China

**Keywords:** melanoma, ligand-receptor pairs, subtyping and scoring model, cancer, genetics

## Abstract

Melanoma is a malignancy of melanocytes, responsible for a high percentage of skin cancer mortality. Ligand-Receptor pairs, a type of cellular communication, are essential for tumor genesis, growth, metastasis, and prognosis. Yet, the role of Ligand-Receptor pairs in melanoma has not been fully elucidated. Our research focused on the function of Ligand-Receptor pairs in melanoma prognosis. We screened 131 melanoma prognosis corresponded ligand-receptor pairs by analyzing the TCGA data of melanoma and the 2293 LR pairs retrieved from the connectomeDB2020 database. And further developed subtypes of melanoma according to the expression of these ligand-receptor pairs by Consensus Clustering. Then we using lasso cox regression and stepwise multivariate regression analysis established a ligand-receptor pairs-based scoring model for the evaluation of melanoma prognosis. Our study demonstrated that the ligand-receptor pairs are vital to the molecular heterogeneity of melanoma, and characterized three different melanoma ligand-receptor pairs subtypes. Among them, the C3 subtype showed a better prognosis, while the C1 subtype exhibited a low prognosis state. And our analysis then found out that this could be related to the differed activation and inhabitation of the cell cycle and immune-related pathways. Using lasso cox regression and stepwise multivariate regression analysis, we further identified 9 key ligand-receptor pairs and established a scoring model that effectively correlated with the prognosis, immune pathways, and therapy of melanoma, showing that the LR.score model was a trustworthy and independent biomarker for melanoma prognosis evaluation. In sum, we found that ligand-receptor pairs are significantly associated with the prognosis and therapy of melanoma. And our ligand-receptor-based scoring model showed potential for the evaluation of melanoma prognosis and immune therapy outcome prediction, which is crucial to the survival for the patients.

## Introduction

Melanoma is the most dangerous type of skin cancer that origins from melanocytes (Guo et al., 2021; Garbe et al., 2022). Although it accounts for only 2% of all skin cancers, over 90% of cutaneous tumor deaths are because of melanoma (Saginala et al., 2021; Garbe et al., 2022). The incidence of melanoma represents for 1.7% of all new primary malignancies diagnoses worldwide, and nearly 57,000 people died from melanoma in 2020 (Sung et al., 2021). Though surgical removal at an early stage can improve the survival rates of melanoma patients, post-surgery tumor metastasizes can cause an unoptimistic prognosis for many patients (Mrazek and Chao, 2014; Matthews et al., 2017; Randic et al., 2021; Parra and Webster, 2022). Because melanoma is a solid tumor embedded with a high mutational burden, which means the cancer cells are able to escape from the immune system (Kalaora et al., 2022; Villani et al., 2022). Thus, the management and prognosis of melanoma can be challenging.

The tumor microenvironment (TME) is a complex system consisting of many heterogeneous cells with different biological features. Intercellular communication within TME can be crucial for tumorigenesis, development, metastasizing, and prognosis (Bonavia et al., 2011; Guo et al., 2021). Moreover, the heterogeneity of each cell type further increases the complexity of tumors, such as involving different clones of tumor cells or various subsets of immune cells (McGranahan and Swanton, 2017).

Ligand-receptor interaction is an essential cellular communication type that is crucial to pharmacological research and tumor progression (Singer, 1992; Ma et al., 2021). The ligands secreted or presented by the cell can interact with the receptor contained target cell, and their connections form a highly connected signal network through different LR pairs (Heldin et al., 2016; Gladka, 2020; Pan et al., 2022). It was first reported in 1971 as vital in human breast cancer, related to the dysregulation of estrogen receptor expression (Olsnes and Pihl, 1974). Later, scientists gradually discovered that LR pairs are involved in disease development mainly through structural/genetic changes and receptor/ligand expression changes (Ma et al., 2021).

Studies have revealed that ligands-receptor pairs affect subtypes and the prognostic outcomes in different kinds of cancers such as glioma (Yuan et al., 2019), colorectal cancer (Lin et al., 2021), Lung adenocarcinoma (Chen et al., 2020), and triple-negative breast cancer (Pan et al., 2022). However, previous LR pairs study in melanoma was limited. A study has revealed the specific Ligand-Receptor interaction between a given cell and a different cell can be vital to the cellular communication of melanoma (Zhou et al., 2017). Yet, more research of LR pairs functions in melanoma regarding the molecular subtypes, clinical features, and prognosis impacts should be investigated. Thus, in this study, we conducted the molecular subtyping based on the LR pairs, and identified nine significant LR pairs gene through lass regression, and further established a scoring model, which could contribute to the melanoma follow-up clinical identification, treatment, prognosis.

## Methods

### RNA-seq data acquisition

The Bulk RNA-Seq data of TCGA-SKCM were downloaded from the TCGA GDC API and a total of 354 metastatic samples were used after screening. The gene expression data of GSE69504 and GSE54467 data were downloaded from the GEO database (Gene Expression Omnibus, https://www.ncbi.nlm.nih.gov/geo/). 186 and 79 metastatic skin melanoma samples, respectively, were finally included after screening. In this study, we take TCGA-SKCM as the training set and GSE69504 and GSE54467 datasets as independent validation sets. Meanwhile, we also included two melanoma immunotherapy data sets (GSE78220 and GSE91061) from the database.

### Data preprocessing

The samples were preprocessed to meet the criteria for further analysis. Samples were excluded based on the following criteria, 1) samples without clinical follow-up information; 2) sample with no survival time; 3) samples without “Status” status; 4) samples with a survival time of fewer than 30 days; After preselection, sample data were converted from Ensembl to Gene symbol. And we take the median value of multiple Gene Symbols expressions.

### GEO data preprocessing

For the GEO dataset, the annotation information of the corresponding chip platform was downloaded and projected the probes to genes accordingly to remove the probes that matched multiple genes. When multiple probes matched a gene, the median was taken as the gene expression value.

Datasets and samples were listed as attachments *.exp.txt, *.cli.txt.

### Ligand-receptor pairs database

Ligand Receptor (LR) pairs were downloaded from the connectomeDB2020 database. A total of 2293 LR pairs were detected, see all_LR_pairs.txt.

### Patient stratification

For each LR pair, a patient was designated “high” if the sum of the gene expressions of the LR pairs was equal to or greater than the median of the sum of the gene expressions of the LR pair of all patients. Otherwise, the patient was designated “low”.

### Survival analysis

The overall survival rate of the cancer patients was used for survival analysis with the “survival” package (version 3.2–11) in R (version 3.6.0). Statistical significance was evaluated by the Peto and Peto modification of the Gehan-Wilcoxon test. The hazard ratio (HR) was calculated by the exponentiated coefficients of the Cox regression model. We performed survival analysis for each cohort and combined the *p*-values from the three cohorts by Edgington’s method using the “sump” function in the”metap” package (version 1.4). Lastly, Storey’s method for multiple testing corrections (https://doi.org/10.1111/1467-9868.00346) was used for multiple testing corrections using the “qvalue” package (version 2.18.0). LR pairs associated with prognosis The LR pairs associated with patient prognosis were determined as follows: 1) Storey’s q-value <0.05 and 2) and HR > 1 (or HR < 1) in all cohorts.

### Calculate correlation of LR pairs

Considering that co-expression of a ligand and its corresponding receptors is necessary for cell-cell communication through secreting signals, thus we subsequently calculated the Spearman’s correlation coefficients for the significant ligand-receptor pairs in the cell communication analysis in all cohorts. Ligand-receptor pairs with Spearman’s correlation coefficient greater than 0.3 (*p*-value <0.05) were used for consistency clustering analysis to identify molecular subtypes.

### Molecular subtypes based on LR pairs

Consensus clustering (Consensus Cluster Plus) was used to construct a consistency matrix and cluster the samples (Wilkerson and Hayes, 2010). Using the significantly correlated ligand-receptor pairs to screen the molecular subtypes. We utilize the km algorithm and “1-Pearson correlation” as the metric distance, and performed 500 bootstraps with each bootstrap including 80% of the training set patients. The number of clusters was set from 2 to 10, and the optimal classification was determined by calculating the consistency matrix and the consistency cumulative distribution function.

### Consensus clustering analysis of cuproptosis-related genes to screen

The “ConsensusClusterPlus” package in R was used to classify SKCM patients into different clusters according to the consensus expression of related genes. The cluster variable (k) was increased from 1 to 3 to find the most appropriate K value. In review and 858 patients were divided into appropriate clusters.

### Gene set enrichment analysis (GSEA) and functional annotation

We use “GSEA” pathway analysis to investigate pathways of different molecular subtypes in biological processes, where all candidate gene sets were collected from the Hallmark database (Liberzon et al., 2015). The clusterProfiler package was used for functional annotation.

### Tumor immune invasion analysis

We used the CIBERSORT algorithm (https://cibersort.stanford.edu/) to quantify the relative abundance of 22 kinds of immune cells in cutaneous melanoma. And the proportion of immune cells was calculated *via* ESTIMATE software.

### Risk model—Generation and validation of the LRI prognostic risk score model

We calculated the risk score of each patient using the following formula: LR.score=(betai×Expi), i refers to the receptor gene expression level, and beta is the coefficient of the gene in the univariate Cox regression. Zscore was further applied. According to the threshold ‘‘0’’, the patients were divided into high and low-risk groups, and the survival curve was drawn by the Kaplan-Meier method for prognostic analysis. The log-rank test was then used to determine the significance of the difference.

### LR. Score and drug sensitivity correlation analysis

Drug sensitivity data, approximately 1000 cancer cell lines, were downloaded from the Genomics of Drug Sensitivity in Cancer (GDSC) (http://www.cancerrxgene.org). We took the antitumor drug AUC in cancer cell lines as the drug response indicator and adopted Spearman correlation analysis to calculate the correlation between drug sensitivity and LR. Rs|>0.2 was also considered and using Benjamini and Hochberg adjusted FDR. FDR<0.05 was then considered to be significantly correlated. Also, we used the pRRophetic package to predict drug response.

### Clinical specimens and real-time quantitative PCR

20 tumor tissues of SKCM patients were obtained from patients diagnosed with SKCM who underwent surgery at Xiangya Hospital of Central South University. All patients signed informed consent prior to use of clinical specimens. The tumor tissues used in this study were approved by the Ethics Committee of Xiangya Hospital, Central South University. Total tissue RNA was extracted with Trizol (Thermo Fisher Scientific) according to the standard protocol. Next, total RNAs were used to synthesize cDNA with HiScript Q RT SuperMix kit (Vazyme, Nanjing, China). Subsequently, Quantitative PCR analyses were performed using SYBR Green qPCR Master Mix (Bimake). GAPDH was used as an endogenous control. The clinical data are listed in Supplementary Table S1.

### Statistical analysis

All statistical analyses were performed in R software (version 4.2.1), and values of *p* < 0.05 were considered statistically significant.

## Result

### Screening of LR pairs associated with patient prognosis

**FIGURE 1:**
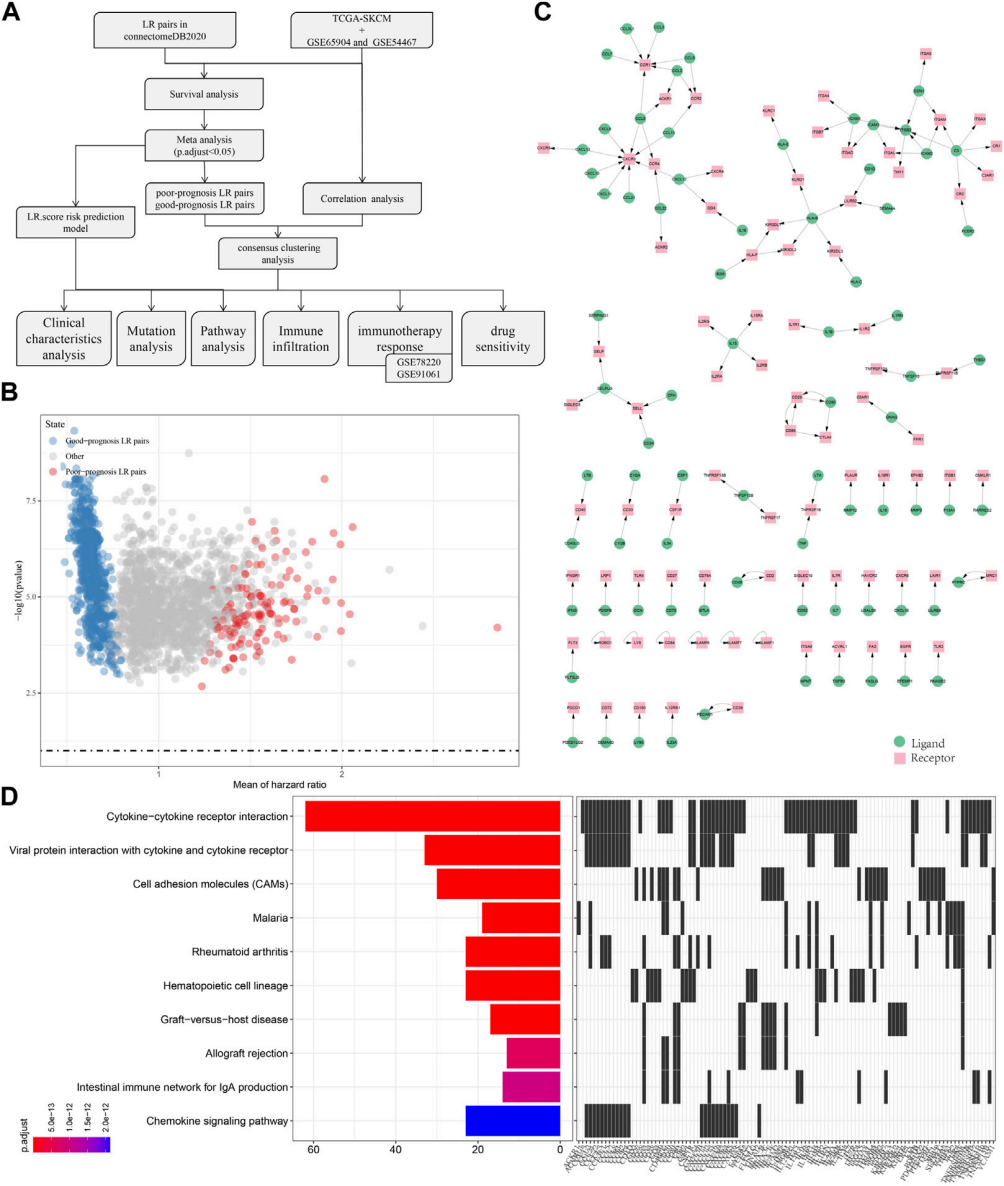
Screen of LR pairs associated with patient prognosis. **(A)** Flow chart of the screening process. **(B)** Volcano plot of LR pairs. **(C)** Interaction network diagram of LR pairs with significant prognosis and significantly correlated expression. **(D)** Top10 KEGG pathway enrichment results of LR pairs with significant prognosis and significantly correlated expression.

To screen for LR pairs associated with cutaneous melanoma prognosis, we included three cutaneous melanoma cohorts, namely TCGA-SKCM, GSE65094, and GSE54467. Firstly, survival analysis of LR pairs on these three cohorts was performed respectively. The analysis results are shown in tcga.LR.HR.res.txt, gse65094.LR.HR.re.txt, and gse54467.LR.HR.res.txt. Then, we carried out a meta-analysis, where the prognostic significance *p*-values of the LR pairs were pooled in the three cutaneous melanoma cohorts, and then corrected for multiple testing. A total of 858 prognostically significant LR pairs was selected, containing 132 poor-prognosis LR pairs and 726 good-prognosis LR pairs, shown as the volcano plot of LR pairs in Figure 1B. Further, we screened the ligand-receptor pairs with significant expression correlation in the three cohorts. And, 131 genes with significant prognosis and receptor-ligand gene expression correlations were finally identified. The interaction network diagram is shown in Figure 1C. Lastly, KEGG pathway enrichment analysis was performed on receptor-ligand genes and found that 131 LR pairs were mainly enriched in Cytokine−cytokine receptor interaction, Viral protein interaction with cytokine and cytokine receptor, Cell molecule adhesion (CAMs), Chemokine signaling pathway equal access.

### Molecular subtypes based on LR pairs

**FIGURE 2:**
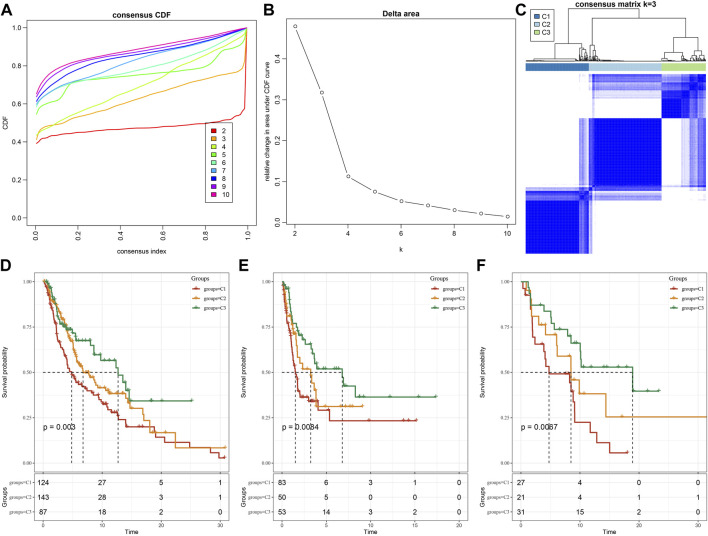
Molecular subtypes based on LR pairs. **(A)** TCGA cohort sample CDF curve. **(B)** TCGA cohort sample CDF Delta area curve. Delta area curve of consensus clustering, indicating the relative change in area under the cumulative distribution function (CDF) curve for each category number k compared with k–1. The horizontal axis represents the category number k and the vertical axis represents the relative change in area under the CDF curve. **(C)** Clustering heat map of TCGA samples when consensus *k* = 3. **(D)** OS time prognosis survival curves of molecular subtypes of TCGA. **(E)** OS time prognosis survival curves of molecular subtypes of GSE65094. **(F)** Three types of subtypes in GSE54467 Prognostic OS time prognostic survival curve.

We further used the screened LR pairs for molecular subtyping and took the sum of the expression values of ligand-receptor genes as the expression intensity of LR pairs. The previously identified 131 LR pairs were significantly correlated and showed a significant prognosis correlation. The 354 skin melanoma samples in the TCGA cohort were clustered by ConsensusClusterPlus, and the optimal number of clusters was determined by the cumulative distribution function (CDF). Then, the CDF Delta area curve showed that three have relatively stable clustering results (Figures 1A, B), and finally we take k = 3 and got three molecular subtypes (Figure 2C; tcga.subtype.txt). Further analysis of the prognostic characteristics of these three subtypes, it is noticeable that they have significant prognostic differences (Figure 2D). In sum, the C3 subtype has a better prognosis effect, while the C1 subtype has a worst prognosis effect. The prognosis effect of the C2 type is between C1 and C3. In addition, we used the same method to have the molecular subtype of the cutaneous melanoma patient cohort of GSE65094, such as gse65094.subtype.txt. It can be observed that these three molecular subtypes have significant differences in prognosis as well (Figure 2E), which is consistent with the training set. This can be also seen in the GSE54467 cohort (Figure 2F, gse54467.subtype.txt). The results suggest that these three molecular subtypes based on ligand-receptor pairs are portable across different study cohorts.

### Comparison of different molecular subtypes with clinical information

**FIGURE 3:**
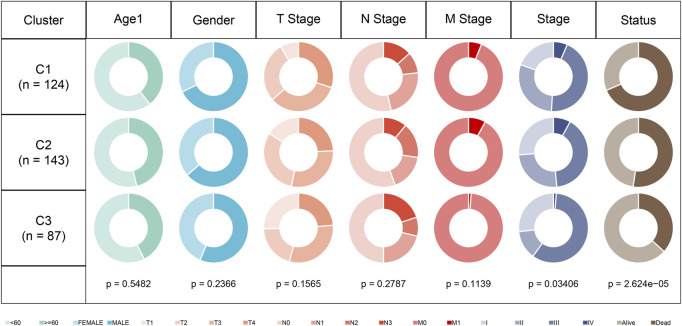
Clinical information distribution of three molecular subtypes in the TCGA cohort.

In the TCGA dataset, we compared the distribution of different clinicopathological features in the previously identified three molecular subtypes. It can be found that there are significant differences among the annual “Stage” of the three molecular subtypes (Figure 3).

### Mutational signatures of different molecular subtypes

**FIGURE 4:**
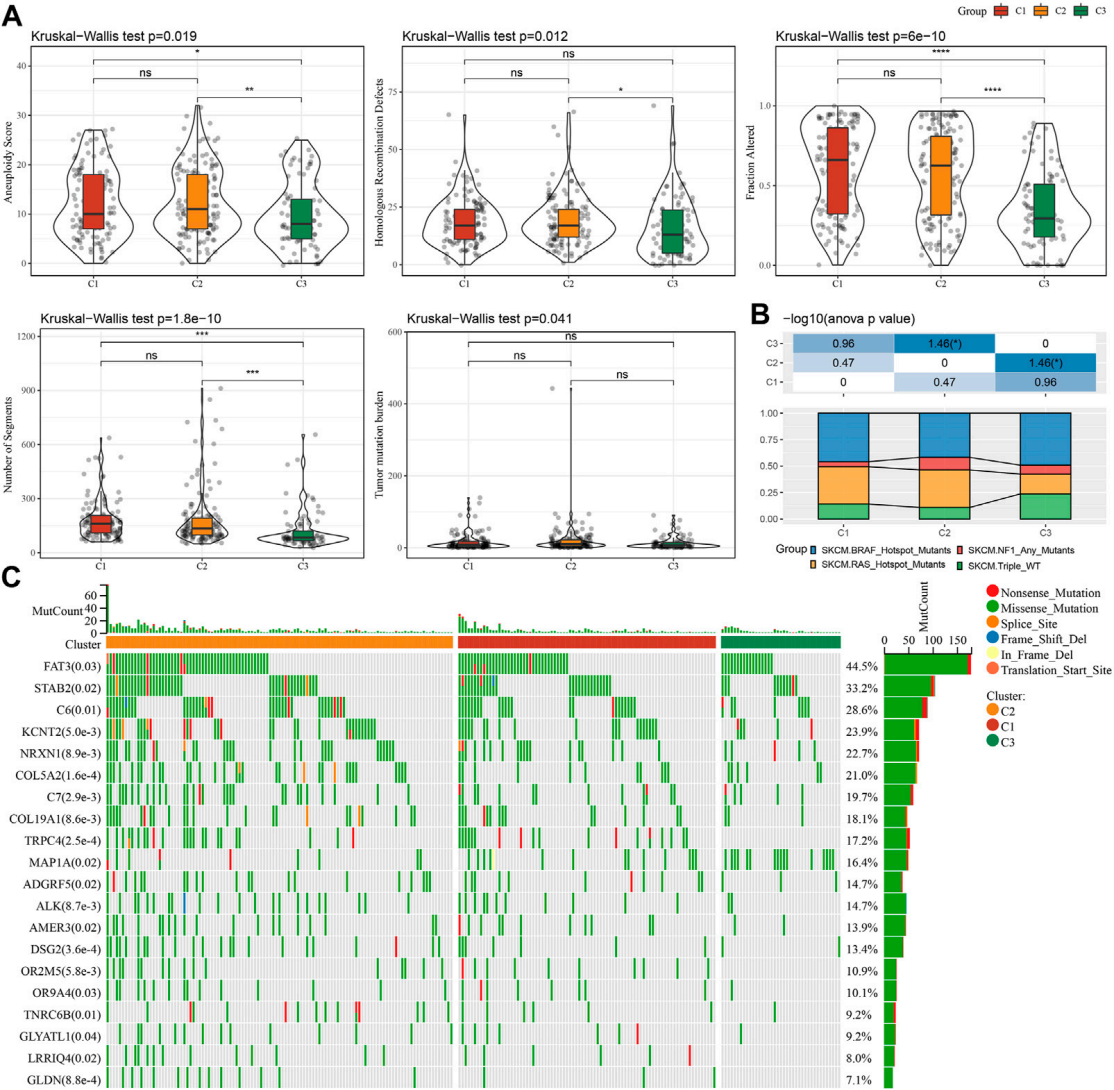
Genomic alterations in molecular subtypes of the TCGA cohort. **(A)** Comparison of Homologous Recombination Defects, Aneuploidy Score, Fraction Altered, Number of Segments, and Tumor mutation burden in the molecular subtypes of the TCGA cohort. **(B)** Comparison of the three molecular subtypes with other molecular subtypes. **(C)** Somatic mutations in three molecular subtypes (chi-square test).

In order to further explore differences in genomic alterations between these three molecular subtypes in the TCGA cohort. We collected molecular characterization information of TCGA-SKCM from a previous pan-cancer study (Thorsson et al., 2018), showing that the Aneuploidy Score, Homologous Recombination Defects, Fraction Altered, Number of Segments, and Tumor mutation burden among these molecular subtypes are not significant. We also compared the relationship between the 4 molecular subtypes, which were mentioned in the previous study (Thorsson et al., 2018), with our three molecular subtypes (Figure 4B). We can find the proportion of “BRAF_Hotspot_Mutants”, “BRAF_Hotspot_Mutants”, “NF1_Any_Mutants” and “RAS_Hotspot_Mutants” in C1 and C2 subtypes were higher than that of C3 subtypes. In addition, we also compared the differences in gene mutations between different molecular subtypes. The top20 genes with significant differences are shown in Figure 4C, indicating that the mutation frequencies of FAT3, STAB2, and others are significantly different among the three molecular subtypes.

### Different molecular subtypes pathway analysis

**FIGURE 5:**
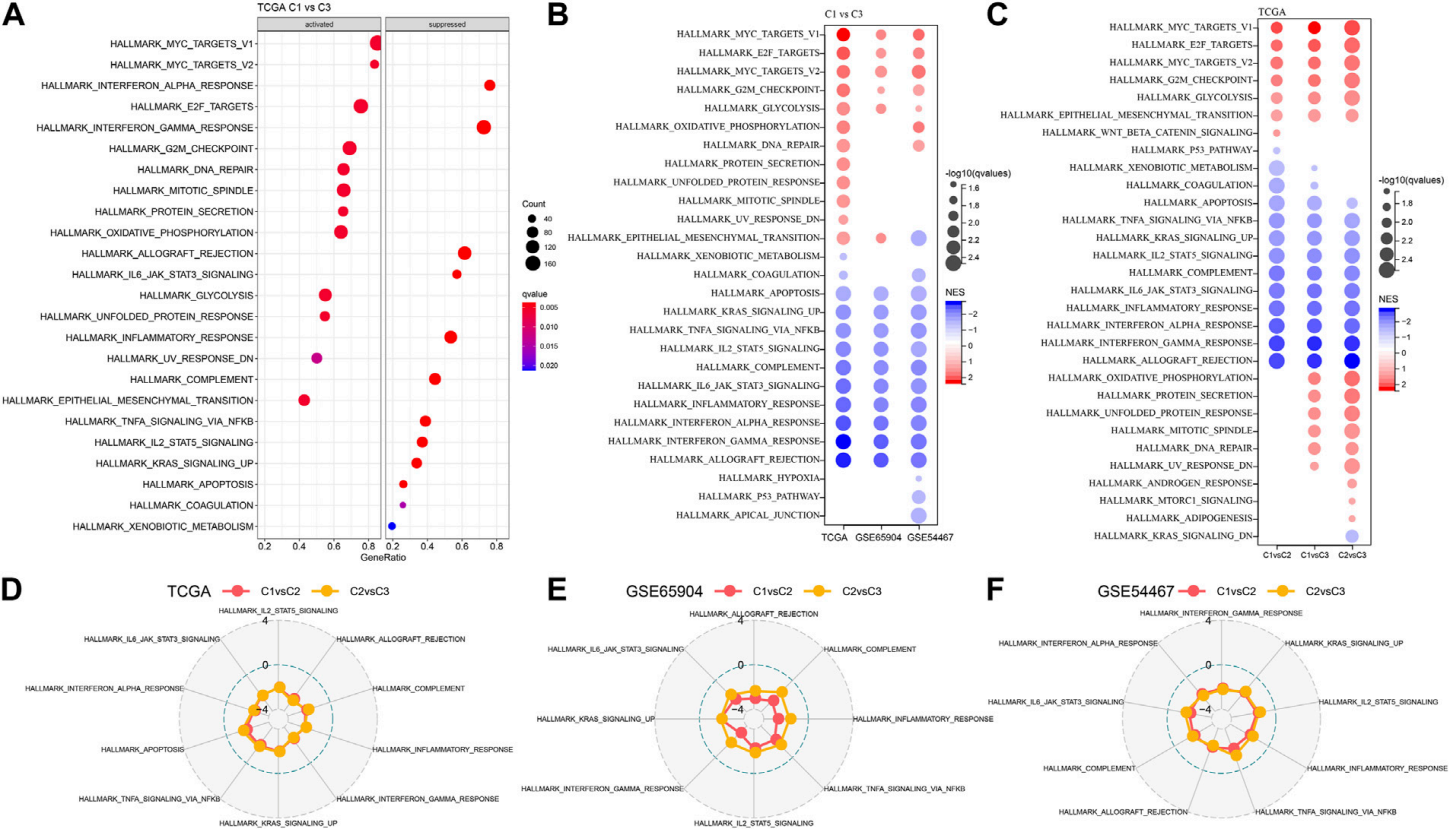
Different molecular subtypes pathway analysis. **(A)** Results of GSEA analysis of C1 vs. C3 in the TCGA-SKCM cohort. **(B)** Bubble plot of GSEA analysis of C1 vs. C3 subtypes in the three cutaneous melanoma cohorts. **(C)** Different molecular subtypes in the TCGA-SKCM cohort. **(D–F)** Radar plots indicating NESs of Hallmark pathways calculated through a gene set enrichment analysis (GSEA) of cluster 1 *versus* cluster 2 and of cluster 2 *versus* cluster 3 in the TCGA cohort, GSE69504 cohort, and GSE54467 cohort.

We further analyzed differentially activated pathways in different molecular subtypes. To identify these pathways, we carried GSEA analysis using all candidate gene sets found in the Hallmark database (Liberzon et al., 2015). And FDR <0.05 were considered as significant enrichment. Noticeably, compared with the C3 subtype in the TCGA cohort, 12 pathways are activated and 12 pathways are inhibited in the C1 subtype (Figure 5A). The activated pathways are mainly cell-cycle-related pathways such as HALLMARK_MYC_TARGETS_V1, HALLMARK_E2F_TARGETS, HALLMARK_MYC_TARGETS_V2, HALLMARK_G2M_CHECKPOINT, etc. While the inhibited pathways are mainly immune-related pathways such as HALLMARK_COMPLEMENT, HALLMARK_INFLAMMATORY_RESPONSE, HALLMARK_INTERFERON_ALPHA_RESPONSE, HALLMARK_INTERFERON_GAMMA_RESPONSE, HALLMARK_ALLOGRAFT_REJECTION, etc. In addition, we also analyzed the mentioned pathways of C1 subtypes in the GSE65094 and GSE54467 cohorts. In general, the activated pathways are mainly the cell cycle-related pathways, and the inhibited pathways are mainly the immune marker pathways (Figure 5B). In addition, we also compared the pathways of different TCGA-SKCM cohorts between each of the three subtypes, C1, C2, and C3 (Figure 5C). It showed that significant enrichment was found in cell cycle and immune-related pathways among the different subtypes analyzed. Through GSEA analysis among different subtypes, C1 patients showed an inhibitory state in immunomodulatory pathways, while cell cycle-related pathways were in an activated state, which reveals that these molecular subtyping-related ligand-receptors may play an important role in the immune microenvironment and cell cycle.

### Immune characterization of different molecular subtypes

**FIGURE 6:**
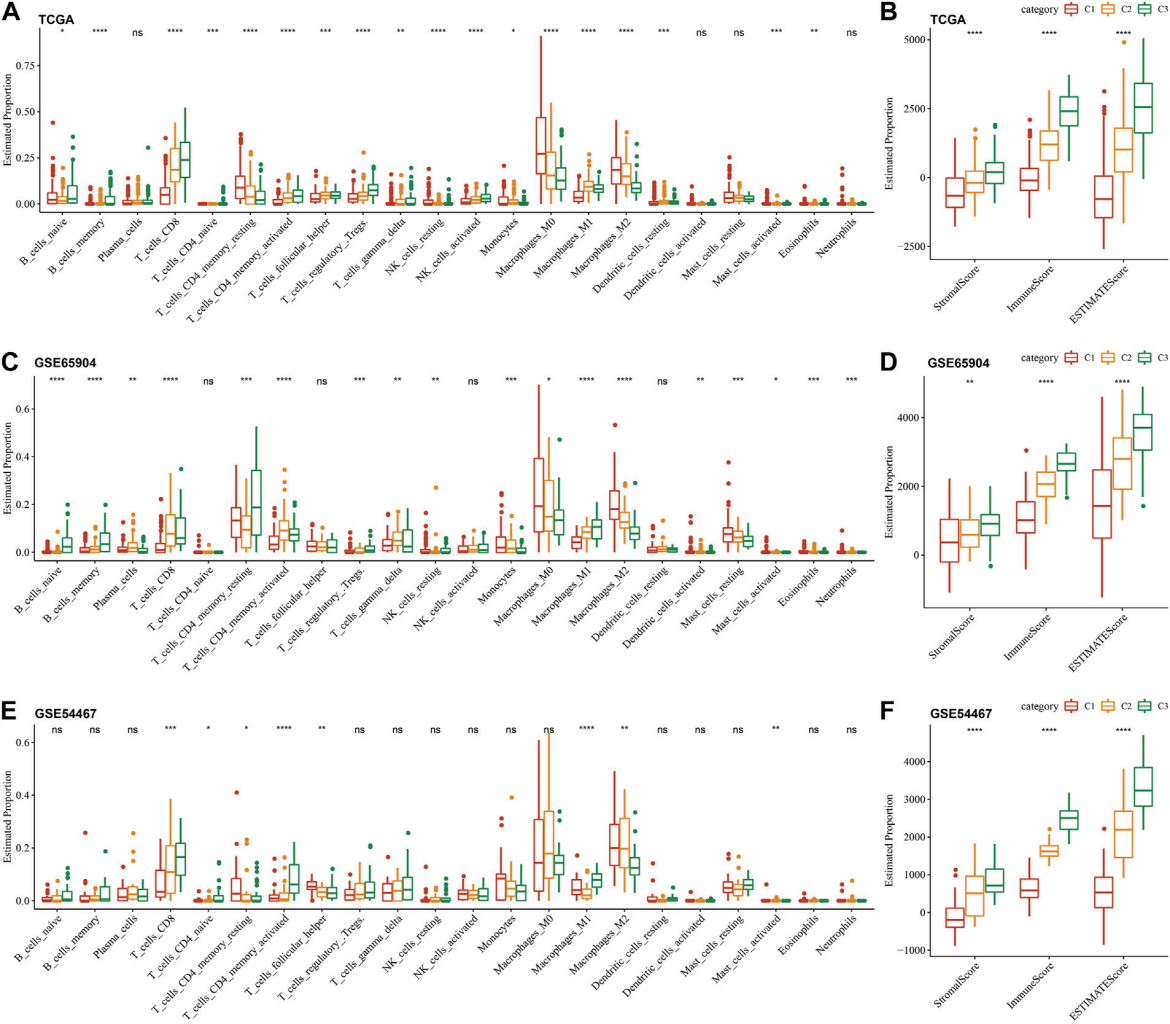
Immune characterization of different molecular sub-types. **(A–F)**: Differences in immune cell infiltration between different molecular subtypes in CIBERSORT and ESTIMATE in the three cutaneous melanoma cohorts.

To further elucidate the differences in the immune microenvironment of three different molecular subtypes, we quantified the relative abundance of 22 immune cells in three cutaneous melanoma cohorts through the CIBERSORT algorithm. The results of the immune cell differences in multiple molecular subtypes in TCGA, GSE65094, and GSE54467 cohorts are shown in Figures 6A, C, E, respectively. We can see that the three molecular subtypes we identified have significant differences in some immune cells. Meanwhile, we also used ESTIMATE to evaluate immune cell infiltration, shown in Figures 6B, D, F. The “ImmuneScore” of the C3 subtype in the TCGA, GSE65094, and GSE54467 cohorts are higher than other subtypes, indicating that the C3 subtype has relatively high immune cell infiltration.

### Scoring model based on ligand-receptor pairs

In previous analyses, we found that molecular subtypes based on LR pairs have distinct mutational landscapes, pathway signatures, and degrees of immune infiltration. And, for the 858 LR pairs with significant meta-analysis results, we further adopted lasso regression to compress them in the TCGA-SKCM cohort to reduce the number of genes in the risk model. The lasso cox regression was performed *via* R package glmnet. As shown in the change trajectory of each independent variable (Supplementary Figure S1A), with the gradual increase of lambda, the number of independent variable coefficients tending to 0 also gradually increases. And we further use 10-fold cross-validation to build the model and analyze the confidence interval under each lambda (Supplementary Figure S1B). It is evident that the model is optimal when lambda = 0.0849. Thus, we choose the 13 LR pairs when lambda = 0.0849 as the target LR pairs. Further, based on the result of these 13 LR pairs in the lasso analysis, we use stepwise multivariate regression analysis. The stepwise multivariate regression analysis adopted the AIC Akaike information criterion, which takes the statistical fit of the model and the number of parameters used for fitting into account. The stepAIC method in the MASS package starts with the most complex model and removes one variable gradually to reduce the AIC. The smaller the value, the better the model, which means that the model obtains sufficient fit with fewer parameters. Finally, we identified 9 LR pairs as key LR pairs, that is “BMP6- > HJV”, “CCL8- > ACKR4”, “DLL3- > NOTCH3”, “DSC3- > DSG3”, “GHRH- > GHRHR” ", “LRRC4B- > PTPRF”, “SEMA4D- > PLXNB1”, “SFRP1- > FZD6”, “UCN- > CRHR1”, the multivariate COX regression coefficient results of these 9 LR pairs are shown in Supplementary Figure S1C.

**FIGURE 7:**
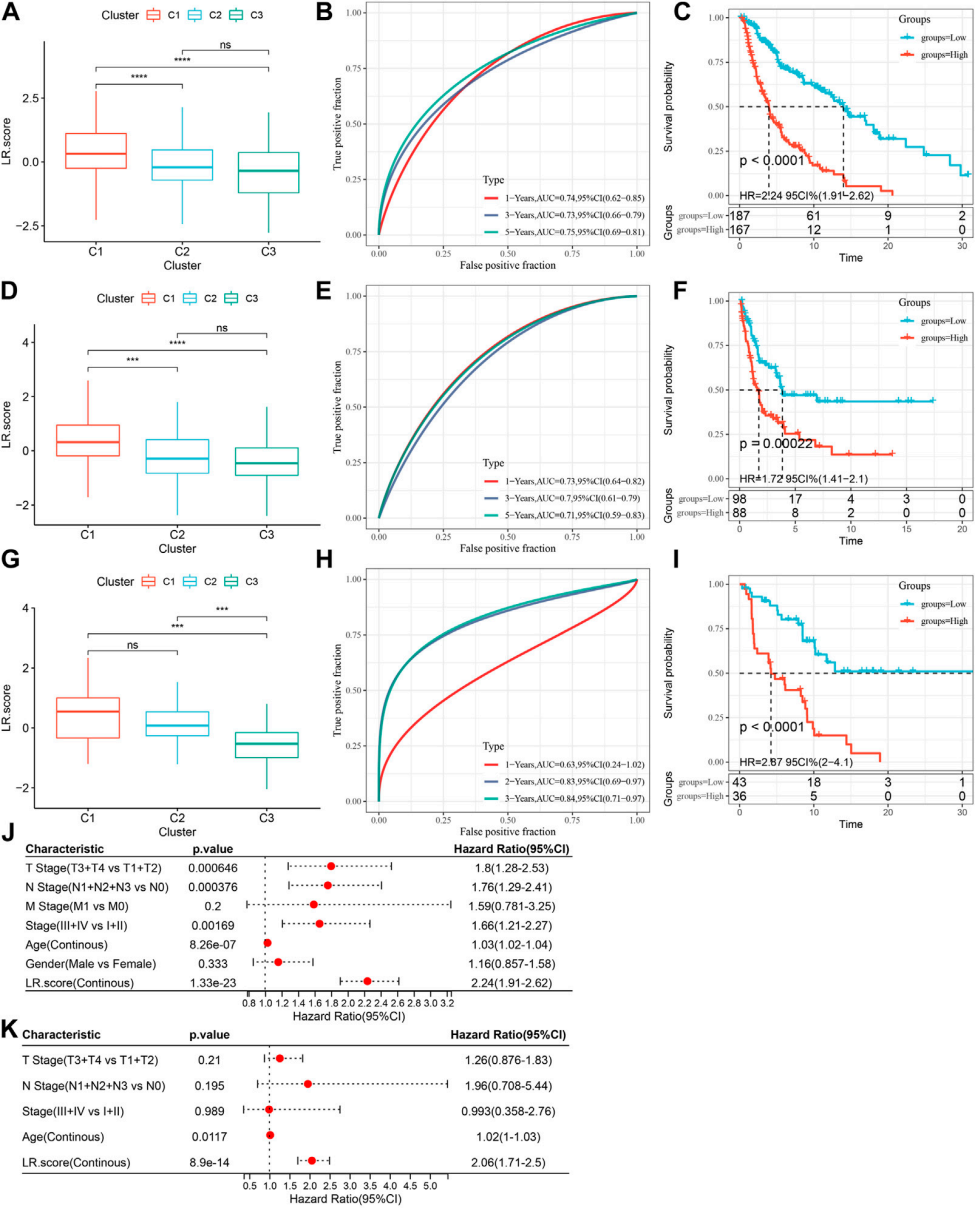
Scoring model based on ligand-receptor pairs **(A)** LR.score differences in multiple LR.score groups in the TCGA-SKCM cohort. **(B)** Survival analysis in the high and low LR.score groups in the TCGA-SKCM cohort. **(C)** The predictive value of LR.score in patients among the TCGA-SKCM cohort (AUC: 0.74, 0.73 and 0.75; 1, 3 and 5-years overall survival). **(D)** LR.score differences in multiple LR.score groups in the GSE65094 cohort. **(E)** LR.score high and low groups in the GSE65094 cohort Survival analysis. **(F)** The predictive value of LR.score in patients among the GSE65094 cohort (AUC: 0.73, 0.70 and 0.71; 1, 3 and 5-years over all survival). **(G)** LR.score differences in multiple LR.score groups in the GSE54467 cohort. **(H)** Survival analysis in high and low LR.score groups of the GSE54467 cohort. **(I)** The predictive value of LR.score in patients among the GSE54467 cohort (AUC: 0.63, 0.83 and 0.84; 1, 3 and 5- years overall survival). **(J)** Univariate Cox regression model analysis, which included the factors of LR.score, patient age, gender, TNM stage and patient outcomes in the TCGA-SKCM. **(K)** Multivariate Cox regression model analysis, which included the factors of LR.score, patient age, gender, TNM stage and patient outcomes in the TCGA-SKCM.

**FIGURE 8:**
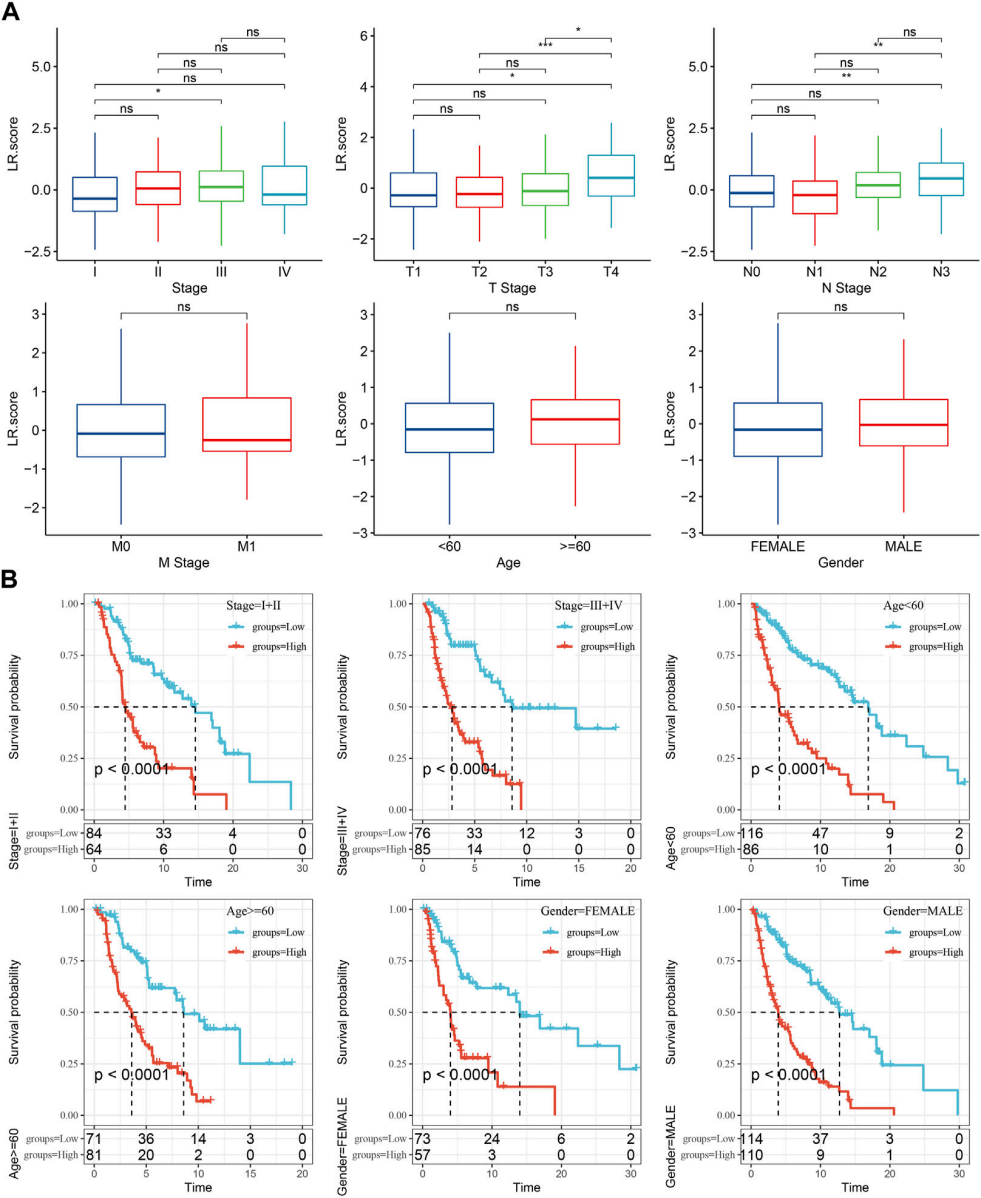
Clinical features of LR.score. **(A)** Distribution differences of LR.score in different clinicopathological features in TCGA-SKCM cohort. **(B)** KM curve between high and low risk groups of LR.score among different clinicopathological groups in TCGA-SKCM cohort.

Then, we constructed an LR pairs scoring model based on these 9 LR pairs to quantitatively analyze the LR pairs pattern of cutaneous melanoma patients, called the LR pairs score. We found that the LR.score of molecular subtype “C1″ was higher than that of subtypes “C2” and “C3” (Figure 7A). We further divided patients into two groups with low and high LR.score scores to further assess the clinical relevance of the LR pairs scores, whose cut-off scores were determined according to the threshold of “0". Patients with low LR.score in the TCGA-SKCM cohort showed a significant survival benefit (Figure 7B; log-rank test, *p* < 0.0001). The AUC of the time-dependent ROC curve of LR.score at 1, 3, and 5 years overall survival time was 0.74, 0.73, and 0.75, respectively (Figure 7C). To test whether LR.score can be used as a suitable independent prognostic factor, we performed univariate and multivariate Cox regression analysis using the clinical characteristics of patients (including age, gender, cytogenetics risk category, and FAB category, etc.). The results showed that LR.score was a reliable and independent prognostic biomarker for evaluating patient prognosis (Figure 7K; HR = 2.06, 95% confidence interval 1.71–2.5, p < 1E-5). The reliability of the LR.score was validated using 186 samples from GSE65094 (Figures 7D–F). Similarly, patients with low LR.score in the GSE65094 dataset also showed a significant survival benefit (Figure 8E; log-rank test, *p* = 0.00022). The AUC of the time-dependent ROC curve for LR.score at 1, 3, and 5-year overall survival time were 0.73, 0.7, and 0.71, respectively (Figure 7F). This can be also found in the GSE54467 cohort (Figures 7G–I). Overall, it suggested that LR.score can reflect the LR pairs pattern of patients with cutaneous melanoma and predict prognosis.

### Clinical features of LR.score

The differences in LR.score within clinicopathological features in the TCGA-SKCM dataset were then analyzed to examine the relationship between LR.score and clinical features of cutaneous melanoma, indicating that higher clinical stage grades were accompanied by higher LR.score scores (Figure 8A). In addition, we also compared whether the LR.score grouping we defined had prognostic differences between different clinicopathological feature groups in the TCGA-SKCM cohort, and the results proved that our grouping also had good effects on different clinical groups, demonstrating the reliability of our risk grouping (Figure 8B).

### Correlation between LR.score and immunity and related pathways

**FIGURE 9:**
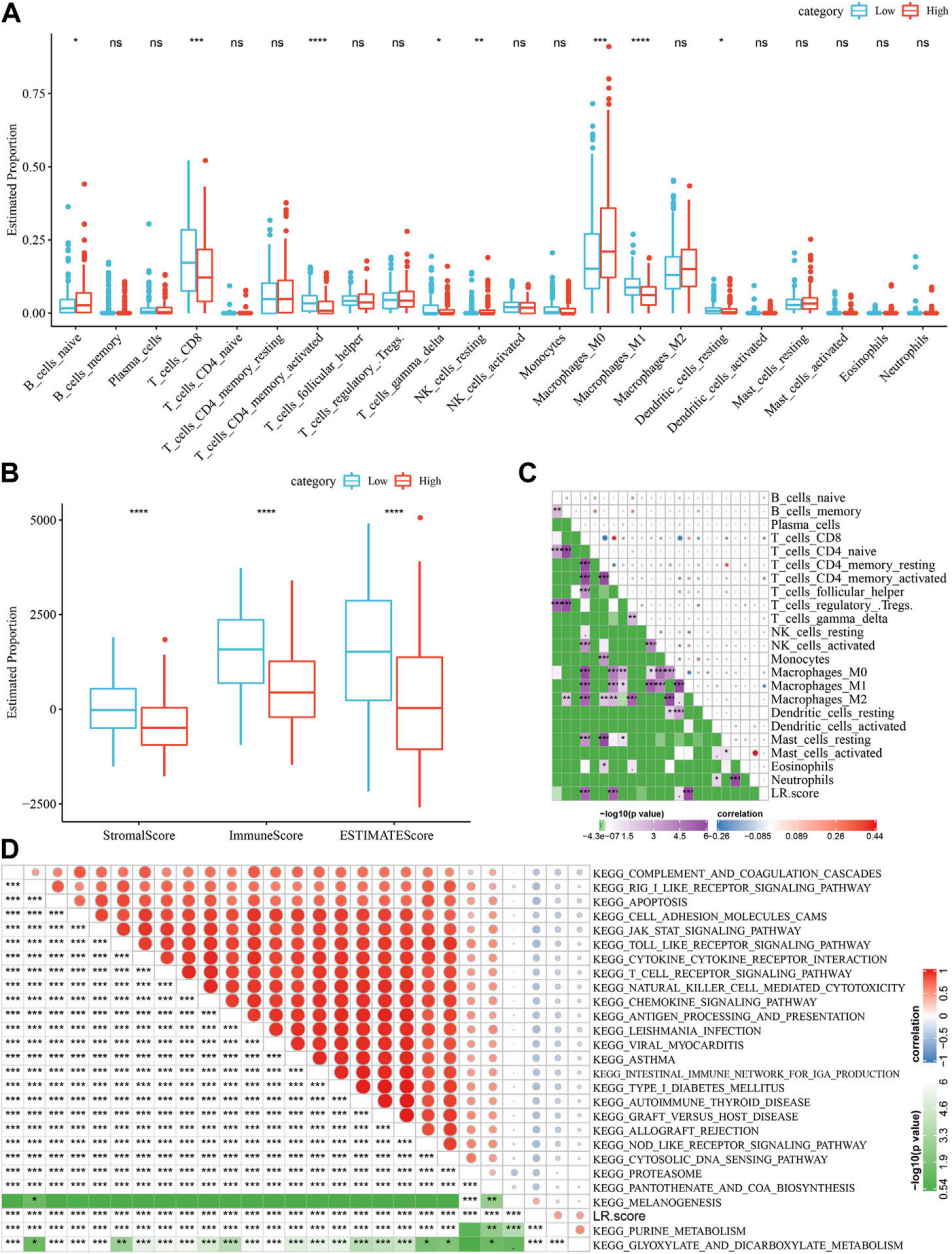
Correlation between LR.score and immunity and related pathways. **(A)** The proportion of immune cells in the TCGA-SKCM cohort. **(B)** The proportion of immune cells in the TCGA-SKCM cohort calculated by ESTIMATE software. **(C)** The correlation between 22 immune cells in TCGA-SKCM and the LR.score. **(D)** KEGG pathway and LR.score in TCGA-SKCM whose correlation with LR.score is greater than 0.4.

Next, we analyzed the differences in the score distribution of 22 immune cells in the LR.score groups in the TCGA-SKCM cohort (Figure 9A). In general, some immune cell scores were significantly different among the LR.score groups, such as B_cells_naive, T_cells_CD8, T_cells_CD4_memory_activated, T_cells_gamma_delta, Macrophages_M1. We also compared the immune infiltration in the LR.score groups and found that the immune ImmuneScore in the low LR.score groups was significantly higher than in the high LR.score groups (Figure 9B). The Pearson correlation coefficient was further used to calculate the correlation between the immune signature index and immune cells to check the relationship among LR.score and 22 immune cell scores in the TCGA-SKCM cohort, showing that T_cells_CD8, T_cells_CD4_memory_activated, Macrophages_M1 were significantly negatively correlated (Figure 9C). In order to reveal the relationship between LR.score and biological functions, we choose the gene expression profiles corresponding to skin melanoma samples in the TCGA-SKCM cohort and used the R software package GSVA to perform a single-sample GSEA analysis (ssgsea) to calculate the different functions of each sample. The correlation between these functions and LR.score were further calculated, and the function with a correlation greater than 0.4 is selected (Figure 9D). The results showed that 24 pathways were negatively correlated with the LR.score of the sample, 2 pathways were positively correlated with the LR.score, and the negatively correlated pathways with the LR.score were mainly immune-related pathways.

### Differences between LR.score model and immunotherapy/chemotherapy

**FIGURE 10:**
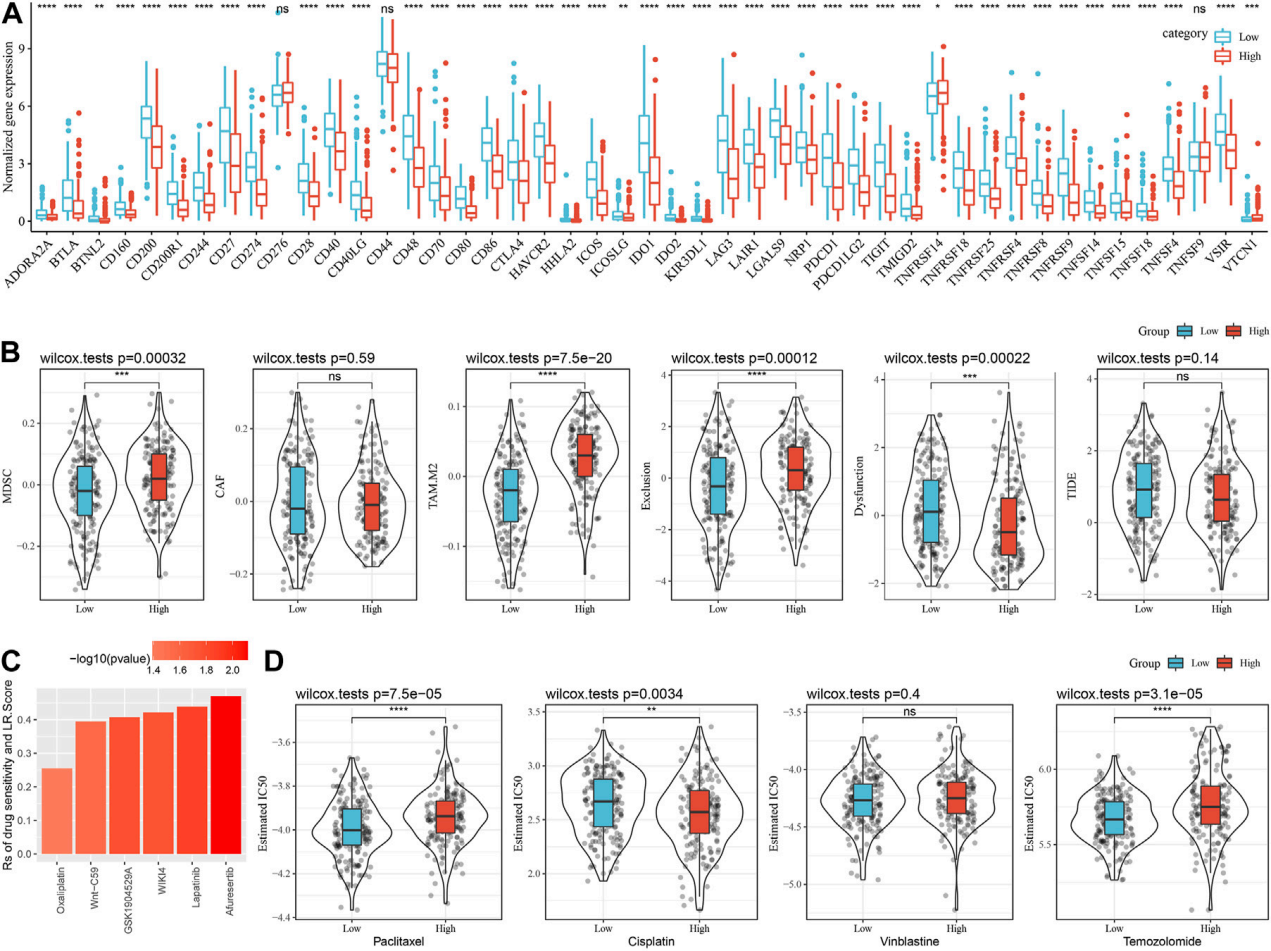
Differences between LR.score model and immunotherapy/chemotherapy. **(A)** Expression of immune expression points between LR.score groups in TCGA-SKCM cohort. **(B)** TIDE analysis results between LR.score groups in TCGA-SKCM cohort. **(C)** LR.score score and Correlation of drug responses in tumor cell lines. **(D)** The box plots of the estimated IC50 for Paclitaxel, Cisplatin, Vinblastine and Temozolomide in TCGA-SKCM.

The response to immunotherapy among the LR.score groups was then analyzed. Firstly, we compared the differences in the expression of immune checkpoints among the LR.score groups. It can be seen that some immune checkpoint genes are differentially expressed in the LR.score groups (Figure 10A). Further, we analyzed the differences between different LR.score groups in immunotherapy, using TIDE (http://tide.dfci.harvard.edu/) software to assess the potential clinical effects of immunotherapy in the previously defined LR.score high and low groups. The higher TIDE prediction score means a higher possibility of immune escape, indicating the less benefit patient can get from immunotherapy. As shown in Figure 10C, the high LR.score group has a lower TIDE score in the TCGA-SKCM cohort, suggesting that the high LR.score group is more likely to benefit from immunotherapy. Meantime, we also compared the differences in the predicted T cell dysfunction score and T cell rejection score among different metabolite subtypes in the TCGA-SKCM cohort and found that the high LR.score group had the highest T cell rejection score.

To further understand the effect of LR.score on drug response, we assessed the relationship between LR.score and tumor cell lines drug response. Spearman correlation analysis was taken and identified 6 pairs of significant correlations between LR.score and drug sensitivity in the Genomics of Cancer Drug Sensitivity (GDSC) database (Figure 6), which showed drug resistance associated with LR.score. In addition, we also adopted the R package pRRophetic to evaluate the chemotherapy response in the high and low LR.score groups in the TCGA-SKCM cohort. After analyzing the effects of several commonly used chemotherapy drugs, Paclitaxel, Cisplatin, Vinblastine, and Temozolomide in LR.score groups, it was found that patients with low LR.score scores were more sensitive to Paclitaxel and Temozolomide. Taken together, these results suggest that LR pairs are associated with drug sensitivity. Therefore, LR.score can be a potential biomarker for establishing appropriate treatment strategies.

### LR.score model to predicts the response to PD-L1 blockade immunotherapy

**FIGURE 11:**
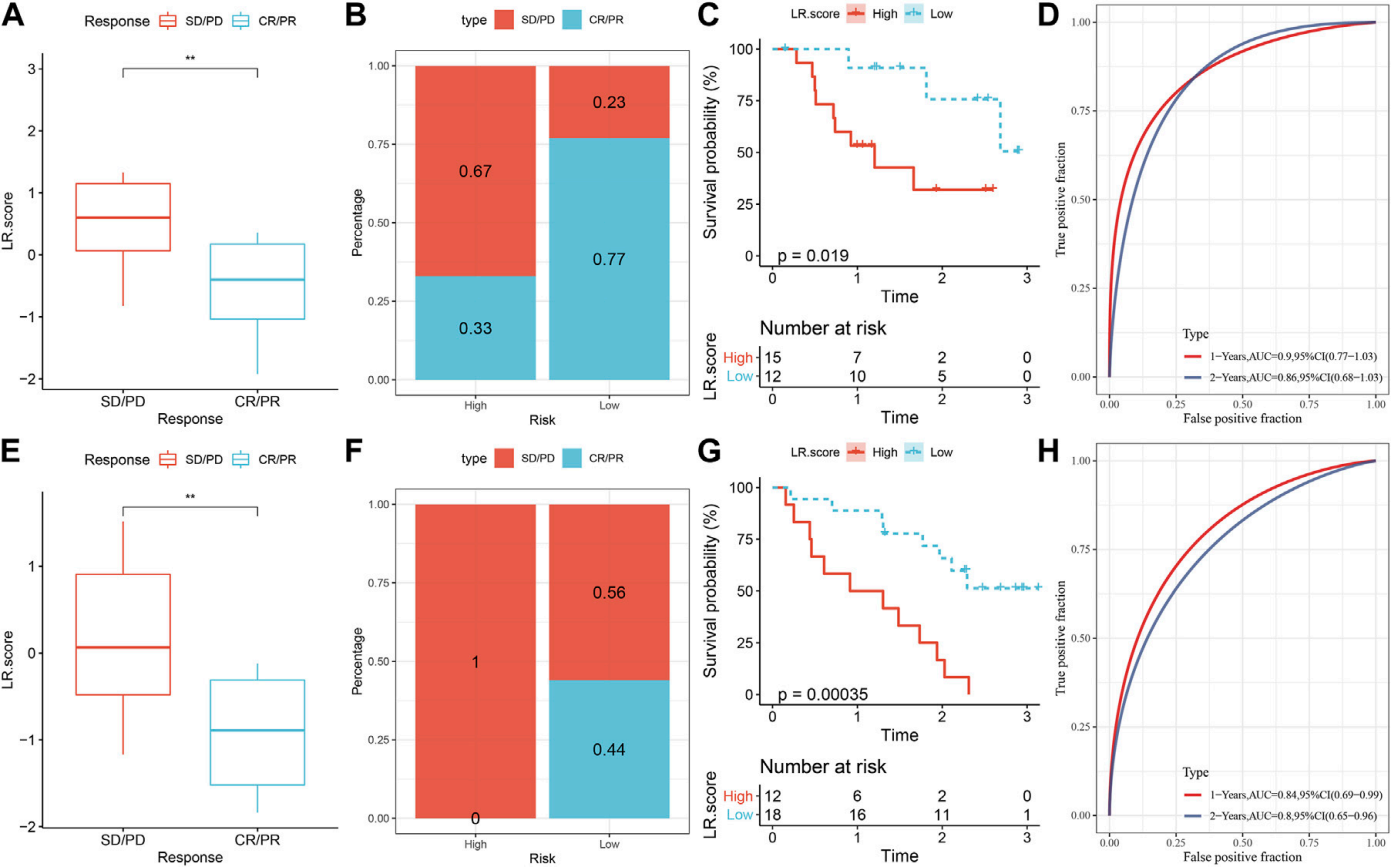
LR.score model to predicts the response to PD-L1 blockade immunotherapy. **(A)** Difference of LR.score between immunotherapy responses in GSE78220 cohort. **(B)** Distribution of immunotherapy response between LR.score groups in GSE78220 cohort. **(C)** Prognostic difference between LR.score groups in GSE78220 cohort. **(D)** The predictive value of LR.score in patients among the GSE78220 cohort (AUC: 0.9 and 0.86; 1 and 2-years overall survival). **(E)** Difference in LR.score between immunotherapy responses in the GSE91061 cohort. **(F)** Immunotherapy response distribution between LR.score groups in GSE91061 cohort. **(G)** Prognostic difference between LR.score groups in GSE91061 cohort. **(H)** The predictive value of LR.score in patients among the GSE91061 cohort (AUC: 0.84 and 0.8; 1 and 2-years overall survival).

Lastly, we analyzed the ability of LR.score to predict patient response to ICB treatment to check the relationship between LR.score and immunotherapy. We noticed that in the anti-PD-1 cohort (GSE78220 and GSE91061), patients with low LR.score showed significant clinical benefits. In the GSE78220 cohort, twenty-seven patients showed varying degrees of response to anti-PD-1 receptor blockers, including complete response (CR), partial response (PR), stable disease (SD), and progressive disease (PD). SD/PD patients had higher LR.score than other types of responders (Figure 11A). The percentage between the low and high LR.score groups also showed that the treatment effect was significantly better for the patients in the low LR.score group (Figure 11B). We then analyzed the survival differences of all GSE78220 samples, showing that the two LR.score scores groups had significant survival differences (Figure 11C) And the AUC of the time-dependent ROC curve of the LR.score was 0.9 and 0.86 for 1 and 2 overall survival time, respectively (Figure 11D). This can be also found in the anti-PD-1 cohort GSE91061.

### Expression of ligand receptor in melanoma

**FIGURE 12:**
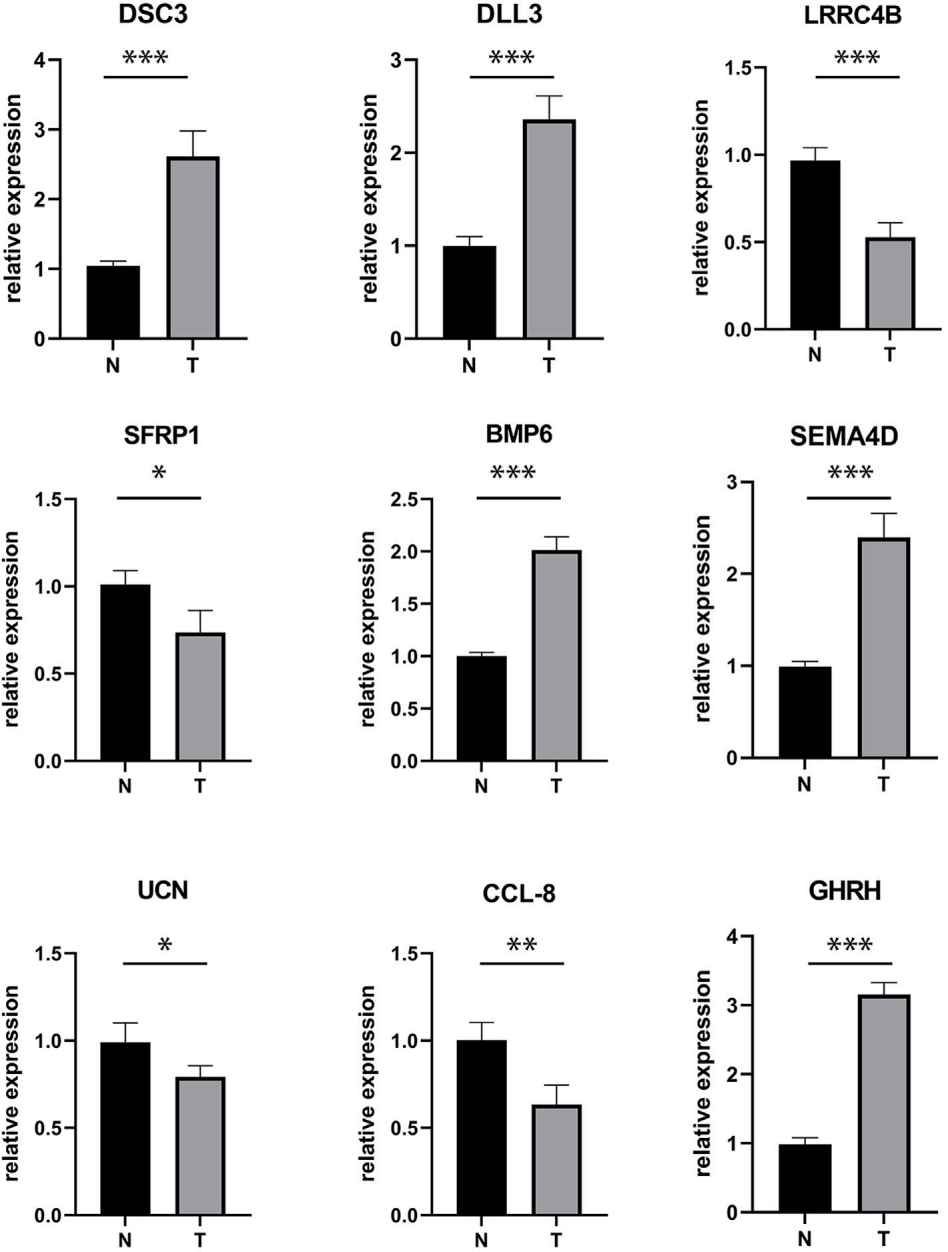
The mRNA expression of LR pairs-related genes in melanoma. N-normal, T-tumor (*p < 0.05, **p < 0.01, ***p < 0.001).

Receptor-ligand interactions are the foundation of all biological events in living cells, and their dysregulation could lead to tumorigenesis (Guryanov et al., 2016). More specifically, specific receptor-ligand pairs can regulate the occurrence and development of tumors. For example, the abnormal activation of the receptor-ligand pairs, DLL3-Notch3, is related to the development of breast cancer, non-small cell lung cancer, and hematological malignancies (Katoh and Katoh, 2020). Similarly, Sema4D is a ligand for Plexin B1 that inhibits c-Met activation and migration and regulates melanocyte survival and growth (Soong et al., 2012). Therefore, in order to further verify the role of the 9 ligand-receptor gene pairs we identified in melanoma, RT-qPCR tests were performed on the expression of the 9 ligands in melanoma samples. The results showed that the expression of DSC3, DLL3, BMP6, SEMA4D, and GHRH are increased (*p* < 0.05) in melanoma, while the expression of LRRC4B, SFRP1, DCN, and CCL-8 decreased in melanoma (*p* < 0.05; Figure 12). These mRNA expression results are consistent with our previous findings, showing that mRNA data analysis can effectively explore the expression of receptor-ligand pairs in melanoma, and provide biological evidence on the differentially expressed ligand-receptor pairs identified by previous results.

## Discussion

In the tumor microenvironment, the communication between different cell types affects the mechanisms of tumorigenesis, progression, therapeutic resistance, and prognosis (Hanahan and Weinberg, 2011). These cell types can communicate through ligand-receptor pairs interactions, where the ligand can be secreted, bound to the receptor in a soluble form, or membrane-bound form, which requires the physical similarity of the two interacting cell types (Ramilowski et al., 2015). The highly connected signal network consisting of multiple LR pairs is vital to the clinical research of cancer (Kim and Cochran, 2017). For instance, one study analyzed scRNA-seq and TCGA RNA-seq data of glioma cells and showed that ligands and receptors notably affect prognostic outcomes (Yuan et al., 2019). Similarly, the research identified colorectal cancer subtypes based on ligand-receptor pairs (LR pairs), indicating that LR pairs are also associated with colorectal cancer immunotherapy response and prognosis (Lin et al., 2021). Lung adenocarcinoma can also find Ligand-receptor interaction atlas within and between tumor cells and T cells (Chen et al., 2020). And LR pairs subtypes also contribute to the prognosis, copy number variation, tumor-infiltrating immune cells, and the immune score of triple-negative breast cancer (Pan et al., 2022). Cell-to-cell interactions (CCIs) through ligand-receptor (LR) pairs in the tumor microenvironment underlie the poor prognosis of pancreatic ductal adenocarcinoma (Suzuki et al., 2021).

Through taking survival analysis and meta-analysis of the relationship between LR pairs and melanoma in three different cohorts, we identified 131 LR pairs, which significantly correlated to the prognosis of melanoma. Based on the expression of these LR pairs, three LR pairs subtypes of melanoma were identified using consensus clustering. In these three subtypes, C3 has a better prognosis and C1 has a poor prognosis. Which could be related to the differential expression of certain genes, such as FAT3, STAB2, etc. It has been reported that the mutation of FAT3, which is related to cytoskeletal and adhesion coding, is related to a negative outcome for melanoma (Yavorski et al., 2017). And tumor metastasis can be prevented by blocking STAB2 function (Hirose et al., 2012). Our further research verified the credibility by analyzing the pathway activation and the abundance of immune cells in these three subtypes. We found that the pathways activated in C1 are mainly Cell cycle-related pathways, and the suppressed pathways in C1 are mainly immune-related pathways. Moreover, we found out that the “ImmuneScore” of the C3 subtype was higher than that of the other molecular subtypes, indicating that the poor prognosis in C1 subtypes could be related to the inhibition of immune modulation.

Then, we identified 9 LR pairs (“BMP6- > HJV” 、"CCL8- > ACKR4” 、"DLL3- > NOTCH3"、"DSC3- > DSG3"、"GHRH- > GHRHR” 、"LRRC4B- > PTPRF"、"SEMA4D- > PLXNB1"、"SFRP1- > FZD6"、"UCN- > CRHR1″) as key LR pairs in melanoma prognosis through Lasso Cox regression and Multiple Stepwise Regression. These 9 LR pairs are reported to play critical roles in melanoma, involving in the occurrence, progression, and metastasis of melanoma. For example, BMP6, showed high expression in melanoma cells and tissues, and the BMP6 deficiency in mice could lead to delayed melanoma tumor onset and decelerated tumor progression (Stieglitz et al., 2019). Cytoplasmic CCL8 also showed rich expression in melanoma tissue, which could induce increased cellular migration in tumor cell line (Yang et al., 2021). DLL3 was found highly expressed in melanoma compared with normal skin (Chen and Yan, 2022), and using Rovalpituzumab tesirine, a DLL3-targeting antibody-drug conjugate, can have antitumor activity in melanoma patients (Mansfield et al., 2021). PTPRF (LRRC4B receptor) is reported as a melanoma biomarker (Sumantran et al., 2015) and confirmed by another study using Genome-wide microarray analysis (Liu et al., 2013). DSC-3 and SFRP1 are biomarkers with suppressive properties, affecting the metastasis and transfection of melanoma (Riker et al., 2008; Mithani et al., 2011). UCN receptor, CRHR1, is involved with cell viability and proliferation (Slominski et al., 2006; Schally et al., 2015). GHRH can be found in melanoma, acting as an autocrine/paracrine growth factor (Schally et al., 2015). And its receptor, GHRHR, is related to the pathogenesis of melanoma (Szalontay et al., 2014). The antagonists of the GHRHR can inhibit proliferation in non-small cell lung cancer and breast cancer (Schally et al., 2015). UCN receptor, CRHR1, is involved with cell viability and proliferation (Slominski et al., 2006). Sema4D, from the immune semaphorin family, is related to angiogenesis and tumor progression and is vital in immune regulation (Lu et al., 2021). Plexin B1, Sema4D receptor, is a tumor-suppressor protein for melanoma, which functions, in part, through inhibition of the oncogenic c-Met tyrosine kinase receptor (Soong et al., 2012). Overall, these 9 key LR pairs are deeply involved with the genesis, progress, and prognosis of melanoma.

Based on the 9 LR pairs, we established an LR pairs scoring model, which could refer to the LR pairs of melanoma patients and predict the prognosis. Our further investigation revealed that the LR pairs score is creditably associated with the clinical grades of the melanoma patients, which is the higher the score, the higher level of their clinical grades. We further confirmed the mRNA expression level with q-PCR. The result is the same with q-PCR. And some immune cell scores are different between LR.score groups. Our research found that the ImmuneScore in the low LR.score score group was significantly higher than that in the high LR.score score group. And the negatively correlated pathways in LR.score were mainly immune-related pathways. The high LR.score group showed the highest T cell rejection score and was less sensitive to the tumor cell lines drugs, such as Paclitaxel and Temozolomide, when compared with the low score group. Taken together, LR.score may be a potential biomarker for establishing appropriate treatment strategies. However, the present study will be more convincing if scRNA-seq data resolved transcriptomics analysis data. Some of GSE data will be used to analysis the result of this research to prove the accuracy of our experiment.

Lastly, we analyzed the relationship between LR. Scores and Immune checkpoint inhibitors therapy. Immune checkpoints are widely studied in oncology research, which are ligand-receptor pairs that inhibit immune response (Ma et al., 2021). Tumor cells can act with the immune checkpoint Programmed Cell Death Protein 1 (PD-1) to suppress T cell function to evade Immunosurveillance (Chen and Mellman, 2013; O Donnell et al., 2019). The reliability of the LR pairs scores to predict immune checkpoint inhibitor (ICI) treatment response was analyzed in the anti-PD-1 cohorts. We noticed that higher LR pair scores were detected in stable disease and progressive disease patients. Moreover, the significantly better clinical benefit of anti-PD-1 therapy was also found in the low LR pair score group, which suggests that the LR pair score model is effective in predicting anti-PD-1 therapy.

## Conclusion

Melanoma is known as a dangerous type of skin cancer. In this study, we firstly took survival analysis and meta-analysis on the TCGA RNA-seq data of melanoma and LR pairs gene data in different cohorts and provided a landscape of the connections between LR pairs and melanoma. We identified three molecular subtypes of melanoma with significant differences in the aspects of clinicopathological features, mutation signatures, pathway activations, and immune signatures. And a scoring model for melanoma based on 9 key LR pairs was constructed, which could predict immune response, drug therapy effectiveness, and prognostic risk of the patients, showing positive potential in both research and clinical treatment.

## Data Availability

The original contributions presented in the study are included in the article/Supplementary Material, further inquiries can be directed to the corresponding author.
